# Ten-Year Surveillance of Microbiological Non-Compliance in Pork: Associations with Season and Slaughterhouse Scale in Lower Northern Thailand

**DOI:** 10.3390/foods15172980

**Published:** 2026-08-25

**Authors:** Roipim Mapongpeng, Orapun Arjkumpa, Sittichai Choosumrong, Rangsun Charoensook, Saowaluk Rungchang, Naruepol Promkuntod, Bertram Brenig, Sonthaya Numthuam

**Affiliations:** 1 Department of Agricultural Science, Faculty of Agriculture Natural Resources and Environment, Naresuan University, Phitsanulok 65000, Thailand; roipimm65@nu.ac.th (R.M.); rangsunc@nu.ac.th (R.C.); 2 Taphan Hin District Livestock Office, Department of Livestock Development, Phichit 66000, Thailand; 3 The 4th Regional Livestock Office, Department of Livestock Development, Khon Kaen 40260, Thailand; arjkumpa@hotmail.com; 4 Department of Natural Resources and Environment, Faculty of Agriculture Natural Resources and Environment, Naresuan University, Phitsanulok 65000, Thailand; sittichaic@nu.ac.th; 5 Division of Food Science and Technology, Faculty of Agro-Industry, Chiang Mai University, Chiang Mai 50100, Thailand; saowaluk.run@cmu.ac.th; 6 Veterinary Research and Development Center (Lower Northern Region), Phitsanulok 65130, Thailand; naruepolp@dld.go.th; 7 Institute of Veterinary Medicine, University of Göttingen, 37077 Göttingen, Germany; bbrenig@gwdg.de

**Keywords:** pig abattoir, food safety surveillance, hazard occurrence, *Salmonella* spp., seasonality, production scale, Thailand

## Abstract

Microbiological non-compliance in pork may indicate deficiencies in slaughterhouse hygiene and pathogen control. This retrospective surveillance study evaluated associations of season and slaughterhouse production scale with microbiological non-compliance in 3103 pork samples collected in Lower Northern Thailand during 2015–2024. Samples were assessed using Department of Livestock Development criteria for aerobic plate count (APC), coliforms, *Escherichia coli*, *Enterococcus* spp., *Staphylococcus aureus*, and *Salmonella* spp. Outcome-specific mixed-effects logistic regression models included calendar year, season, production scale, and province as fixed effects and slaughterhouse as a random intercept. *Salmonella* spp. was the most frequent non-compliance endpoint (40.90%), followed by APC (36.61%). Compared with winter, the rainy season was associated with higher odds of coliform (adjusted odds ratio [AOR] = 1.75, 95% CI: 1.23–2.50) and *Salmonella* spp. non-compliance (AOR = 1.51, 95% CI: 1.20–1.91). High-scale slaughterhouses had higher odds of coliform (AOR = 2.61, 95% CI: 1.21–5.64), *E. coli* (AOR = 2.39, 95% CI: 1.22–4.69), and *Salmonella* spp. non-compliance (AOR = 2.02, 95% CI: 1.17–3.48) than low-scale facilities. These findings support seasonally targeted, risk-based surveillance and continued direct pathogen testing alongside hygiene indicators.

## 1. Introduction

Animal-source foods, including meat, are essential sources of high-quality nutrients; however, their production and consumption are inherently linked to significant food safety risks [1]. Globally, foodborne diseases affect an estimated 600 million people annually, resulting in approximately 420,000 deaths, with Southeast Asia bearing a disproportionately high burden of these cases [2,3]. Pork is of particular food-safety importance because pigs can harbor microorganisms that may contaminate carcasses and meat during production and slaughter [4,5,6,7]. Foodborne bacteria associated with pork include *Salmonella* spp., pathogenic *Escherichia coli*, and *Staphylococcus aureus*, among others [8]. Effective control therefore requires coordinated hygiene, handling, surveillance, and official-control measures throughout the pork production chain [9].

Microbial contamination may occur at several stages of slaughter and processing through fecal leakage, contaminated equipment and contact surfaces, cross-contamination, and inadequate handling or sanitation [4,10,11]. *Salmonella* spp. may enter slaughterhouses in colonized animals and subsequently disseminate during processing [12,13,14,15]. *E. coli* and coliforms are commonly used as indicators of fecal contamination and process-hygiene deficiencies, whereas *S. aureus* may originate from animals, personnel, or the processing environment [16,17,18,19,20]. Aerobic plate count and other hygiene indicators provide information on the overall microbiological condition of meat and the effectiveness of sanitation and process control, but they do not directly establish the absence of specific pathogens [21,22,23,24,25].

In addition to hygiene performance within slaughterhouses, operational characteristics may influence contamination risk. Differences in production scale can affect infrastructure, staffing, throughput, sanitation consistency, and opportunities for cross-contamination [26,27,28]. Seasonal conditions, including variation in temperature, humidity, rainfall, and water management, may further influence bacterial survival and transmission. Previous studies have reported seasonal or environmental variation in foodborne microorganisms along pork and slaughterhouse production systems, although the direction and magnitude of these associations have differed among settings [29,30,31]. Such variability may reflect differences in climate, facility characteristics, slaughter practices, surveillance design, and the microorganisms evaluated.

Despite the recognized importance of both environmental and production-related influences, available evidence remains limited in two important ways. First, many studies rely on short-term observations [21,32,33,34]. Second, many focus on specific pathogens rather than evaluating a broader microbiological profile that includes both hygiene indicators and pathogens [35,36]. Consequently, long-term patterns of microbiological non-compliance under routine slaughterhouse surveillance, particularly in tropical settings, remain insufficiently characterized.

In Thailand, the Department of Livestock Development (DLD) conducts routine microbiological surveillance of pork from slaughterhouses, including testing for aerobic plate count, coliforms, *E. coli*, *Enterococcus* spp., *S. aureus*, and *Salmonella* spp. National livestock-development strategies have supported monitoring programs intended to assess compliance with microbiological criteria and strengthen official control of slaughterhouses [37]. The resulting surveillance records provide an opportunity to examine microbiological non-compliance over an extended period under routine field conditions. Accordingly, this study aimed to (i) describe the occurrence, provincial variation, and annual temporal patterns of microbiological non-compliance in pork from slaughterhouses collected during 2015–2024 and (ii) evaluate the associations of season and slaughterhouse production scale with microbiological non-compliance in Lower Northern Thailand.

## 2. Materials and Methods

### 2.1. Study Area and Study Design

This study was a retrospective analysis of routine microbiological data collected through the Department of Livestock Development surveillance programme from pig slaughterhouses in Lower Northern Thailand between 2015 and 2024. The study area comprised nine provinces: Phichit, Phitsanulok, Uttaradit, Sukhothai, Tak, Kamphaeng Phet, Phetchabun, Nakhon Sawan, and Uthai Thani.

**Figure 1:**
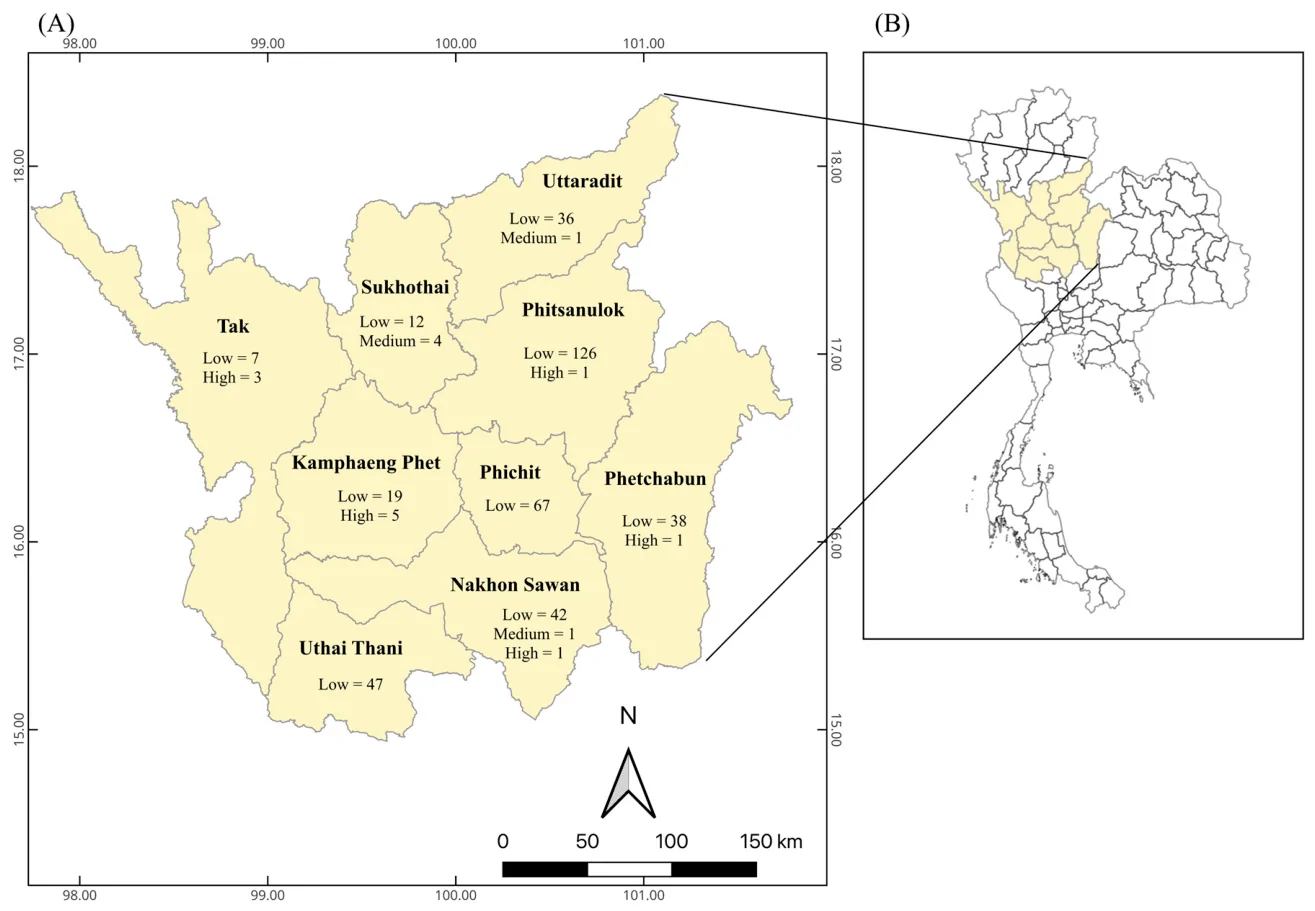
Study area and distribution of participating slaughterhouses by production scale across nine provinces in Lower Northern Thailand, 2015–2024. (**A**) Nine study provinces in Lower Northern Thailand. Numbers indicate the number of surveyed low-, medium-, and high-scale slaughterhouses in each province. (**B**) Location of the study area within Thailand.

The surveillance dataset included 411 unique slaughterhouses that contributed at least one pork sample during the study period. Based on DLD production-capacity criteria, these facilities were classified as low-scale (n = 394), medium-scale (n = 6), or high-scale (n = 11) slaughterhouses. All facilities operated under the supervision of the DLD, Thailand. Figure 1 shows the location of the nine study provinces and the distribution of participating slaughterhouses by production scale.

The unit of analysis was an individual pork sample. Microbiological non-compliance was evaluated separately for aerobic plate count (APC), coliforms, *Escherichia coli*, *Enterococcus* spp., *Staphylococcus aureus*, and *Salmonella* spp.

### 2.2. Data Source, Sample Collection, and Study Population

Pork samples were collected by trained DLD personnel as part of routine slaughterhouse surveillance in Lower Northern Thailand. Surveillance activities were conducted under annual national and provincial monitoring plans designed to support the assessment of slaughterhouse hygiene and microbiological compliance. Provincial livestock offices coordinated with the responsible DLD regional laboratory to establish annual sampling plans and identify slaughterhouses for surveillance. Consequently, the number and identity of participating slaughterhouses were not fixed across calendar years and could vary according to annual surveillance targets, provincial operational plans, and the operating status of individual facilities. The surveillance data therefore represent routine administrative monitoring rather than probability-based sampling of all slaughterhouses in the study area.

The overall surveillance framework was broadly consistent with the nationwide DLD meat-monitoring approach previously described by Klaharn et al. [21], although the present study included routine surveillance records collected over a 10-year period within Lower Northern Thailand. Sample collection, handling, and transportation followed the applicable national monitoring procedures and DLD official guidance [38]. Pork samples consisted of at least 300 g of lean meat without skin or visible fat, including cuts such as tenderloin or ham. Following collection, each sample was placed in an appropriate sterile container, labelled with the required sample information, and maintained under cold-chain conditions below 4 °C during transportation. Samples were transported to the responsible DLD regional laboratory within 24 h using insulated containers with either ice mixed with coarse salt at a ratio of 6:1 or dry ice. All samples were accompanied by the official meat and livestock-product sample submission form and submitted to the Regional Veterinary Research and Development Center in the Lower Northern Region for microbiological analysis.

A surveillance visit was defined as the collection of one or more pork samples from a single slaughterhouse on a given reporting date. The analytic dataset comprised 3103 pork samples collected during 2718 surveillance visits between 2015 and 2024. The 411 slaughterhouses represented unique facilities contributing at least one pork sample during the study period; the number of participating slaughterhouses varied by calendar year. Annual distributions of surveillance visits, participating slaughterhouses, and pork samples by slaughterhouse production scale are presented in Supplementary Table S1. Samples were eligible for inclusion when microbiological test results and corresponding information on collection date, province, slaughterhouse production scale, and slaughterhouse identifier were available.

### 2.3. Microbiological Analysis

Pork samples were analyzed at DLD Regional laboratory centers for APC, coliforms, *E. coli*, *Enterococcus* spp., *S. aureus*, and *Salmonella* spp. in accordance with DLD laboratory procedures. APC was quantified according to the U.S. Food and Drug Administration Bacteriological Analytical Manual (FDA BAM) Online, Chapter 3 (2001) [39]. Coliforms and *E. coli* were analyzed according to FDA BAM Online, Chapter 4 (2013) [40]. *Enterococcus* spp. were examined according to Nordic Committee on Food Analysis Method No. 68, 5th edition (2011) [41], and *S. aureus* was assessed using ISO 6888-1:1999 [42].

Detection of *Salmonella* spp. followed the ISO method applicable under DLD laboratory procedures at the time of analysis. Samples collected before the adoption of ISO 6579-1:2017 [43] were analyzed according to ISO 6579:2002 [44], whereas subsequent samples were analyzed according to ISO 6579-1:2017.

Quantitative results for APC, coliforms, *E. coli*, *Enterococcus* spp., and *S. aureus* were expressed as log_10_ CFU/g. *Salmonella* spp. was assessed qualitatively as detected or not detected in 25 g of sample. The DLD microbiological criteria are presented in Table 1 [38]. The microbiological criteria used to classify samples as compliant or non-compliant remained unchanged throughout the 2015–2024 study period.

### 2.4. Definition of Microbiological Non-Compliance

Samples were classified as compliant or non-compliant according to the DLD microbiological criteria for livestock products (Table 1). For APC, coliforms, *E. coli*, *Enterococcus* spp., and *S. aureus*, samples with counts exceeding the corresponding DLD limit were classified as non-compliant. For *Salmonella* spp., detection in 25 g of pork was classified as non-compliant.

For statistical analyses, compliance status was treated as a binary outcome for each microbiological endpoint, coded as 0 for compliant and 1 for non-compliant.

### 2.5. Study Variables

Calendar year, season, slaughterhouse production scale, and province were included as explanatory variables. Calendar year was defined according to the sample collection date and was categorized from 2015 to 2024. Province was classified into nine categories corresponding to the study area.

Sampling dates were classified into three seasonal periods: summer (February–May), rainy season (June–October), and winter (November–January), with reference to the general seasonal context of Thailand described by the Thai Meteorological Department (TMD). This operational classification was applied consistently to all samples according to their recorded collection dates.

Slaughterhouses were categorized by daily slaughter capacity as low-scale (≤50 pigs/day), medium-scale (51–100 pigs/day), or high-scale (>100 pigs/day) facilities. This classification was consistent with that used in a nationwide survey of pig slaughterhouses in Thailand [21].

A unique slaughterhouse identifier was retained to account for clustering of samples collected from the same facility.

### 2.6. Statistical Analysis

Quantitative bacterial counts were expressed as log_10_ CFU/g and summarized graphically using boxplots. Microbiological non-compliance was summarized as the number and percentage of non-compliant samples overall and by province, calendar year, season, and slaughterhouse production scale. Unadjusted sample-level proportions and Wilson 95% CIs were reported for descriptive purposes, whereas adjusted associations were estimated using mixed-effects models accounting for slaughterhouse-level clustering.

Separate multivariable mixed-effects logistic regression models were fitted for each microbiological endpoint: APC, coliforms, *E. coli*, *Enterococcus* spp., *S. aureus*, and *Salmonella* spp. non-compliance. Calendar year, season, slaughterhouse production scale, and province were included as fixed effects, and slaughterhouse ID was included as a random intercept to account for correlation among samples collected from the same facility. The reference categories were 2015 for calendar year, winter for season, low-scale for slaughterhouse production scale, and Phichit for province. Adjusted odds ratios (AORs), 95% CIs, and two-sided *p*-values were reported. Statistical significance was defined as *p* < 0.05.

Model performance and diagnostics were assessed using convergence warnings, singular-fit diagnostics, slaughterhouse-level random-intercept variance, the intraclass correlation coefficient (ICC), and marginal and conditional R^2^. These results are presented in the Supplementary Materials. Because the distribution of slaughterhouses across production-scale categories was highly unbalanced, the numbers of slaughterhouses, pork samples, and non-compliant samples were summarized by production scale in Supplementary Table S2, and production-scale associations were interpreted cautiously.

As a sensitivity analysis, slaughterhouses were grouped into high-scale and non-high-scale categories, with low- and medium-scale facilities combined in the non-high-scale category. The model included the same fixed effects and slaughterhouse-level random-intercept structure as the primary analysis.

All analyses were performed using R version 4.5.0 [45].

### 2.7. Ethical Statement

Ethical review and approval were not required because this study was a secondary analysis of routine DLD surveillance data derived from pork samples and did not involve live animals, human participants, experimental interventions, or identifiable personal information.

## 3. Results

### 3.1. Distribution of Quantitative Microbiological Counts in Pork Samples

**Figure 2:**
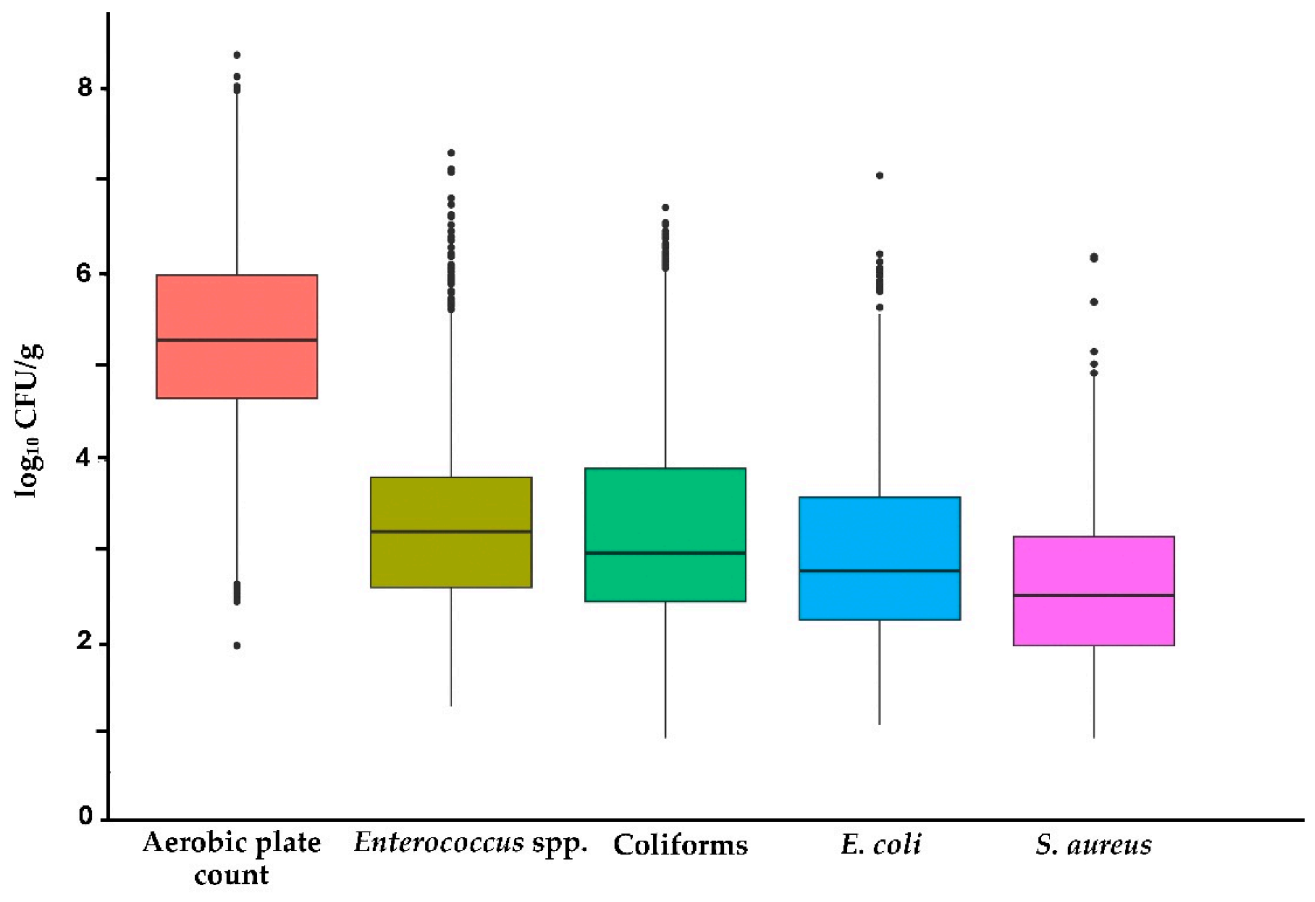
Distribution of quantitative microbiological counts in pork samples collected from slaughterhouses in Lower Northern Thailand during 2015–2024 (n = 3103 per endpoint). Boxes represent the interquartile range, horizontal lines indicate medians, whiskers indicate the spread of non-outlying observations, and points represent outliers.

A total of 3103 pork samples collected from slaughterhouses in nine provinces of Lower Northern Thailand during 2015–2024 were included in the analysis. The distribution of slaughterhouses and pork samples by production scale and province is presented in Supplementary Table S3. Quantitative bacterial loads varied substantially across the measured bacterial groups (Figure 2). Aerobic plate count (APC) showed the highest median counts and generally higher values than coliforms, *Enterococcus* spp., *E. coli*, and *S. aureus*. Outlying observations above the upper whiskers were present for all quantitative endpoints, indicating that some pork samples had markedly elevated bacterial counts. These distributions demonstrate heterogeneity in microbiological contamination among individual pork samples. *Salmonella* spp. was not included in the quantitative distribution because non-compliance was defined categorically as detection in 25 g of pork rather than by a CFU-based threshold.

### 3.2. Overall and Provincial Proportions of Non-Compliance Pork Samples

The proportion of non-compliant pork samples varied across microbiological endpoints and provinces (Table 2). Across all 3103 samples, non-compliance was highest for *Salmonella* spp. (40.90%, n = 1269), followed by aerobic plate count (APC; 36.61%, n = 1136), *Enterococcus* spp. (31.68%, n = 983), *E. coli* (30.20%, n = 937), *S. aureus* (19.85%, n = 616), and coliforms (17.53%, n = 544).

Crude non-compliance proportions varied substantially among provinces (Table 2). APC non-compliance ranged from 18.45% in Sukhothai to 45.45% in Tak. Nakhon Sawan had the highest proportions of non-compliant samples for coliforms (26.58%), *E. coli* (46.58%), *Enterococcus* spp. (42.47%), and *Salmonella* spp. (53.42%), whereas Kamphaeng Phet had the lowest proportion for *Salmonella* spp. (34.93%). Sukhothai had the lowest proportions for coliforms (4.76%) and *Enterococcus* spp. (14.88%), whereas Uttaradit had the lowest proportion for *E. coli* (12.40%). For *S. aureus*, the proportion of non-compliant samples ranged from 14.40% in Uttaradit to 23.98% in Uthai Thani.

### 3.3. Annual Temporal Patterns in Bacterial Non-Compliance

**Figure 3:**
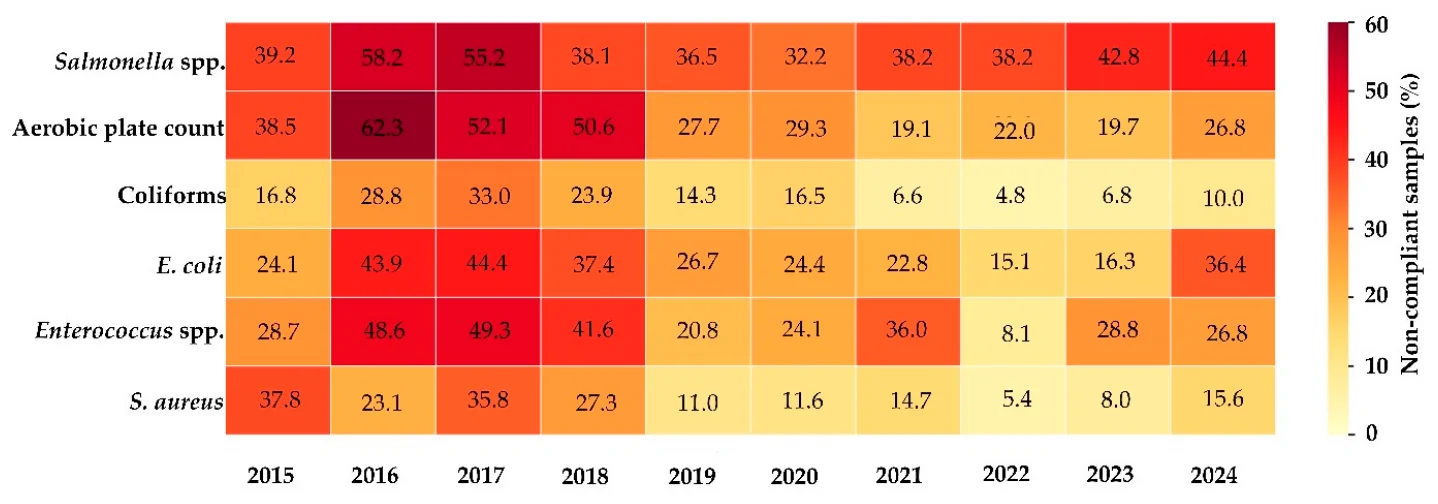
Annual proportions of non-compliant pork samples by microbiological endpoint in slaughterhouses in Lower Northern Thailand, 2015–2024. Heatmap cells show the percentage of samples exceeding Department of Livestock Development microbiological criteria for each endpoint by calendar year.

The annual proportions of microbiological non-compliance varied across the 10-year surveillance period (Figure 3). For APC, coliforms, *E. coli*, *Enterococcus* spp., and *S. aureus*, non-compliance was generally higher during 2015–2018 than in subsequent years, although the timing of the highest annual proportion differed by endpoint. APC non-compliance peaked in 2016 (62.3%), whereas coliforms, *E. coli*, and *Enterococcus* spp. reached their highest annual proportions in 2017 at 33.0%, 44.4%, and 49.3%, respectively. *S. aureus* non-compliance was highest in 2015 (37.8%).

After relatively low values in 2021–2022, non-compliance in 2024 was higher than in 2022 for all five quantitative endpoints. APC increased from 22.0% to 26.8%, coliforms from 4.8% to 10.0%, *E. coli* from 15.1% to 36.4%, *Enterococcus* spp. from 8.1% to 26.8%, and *S. aureus* from 5.4% to 15.6%.

Unlike the other endpoints, *Salmonella* spp. showed a distinct temporal pattern, with consistently high non-compliance proportions throughout the study period. Non-compliance increased from 39.2% in 2015 to a peak of 58.2% in 2016 before declining to 32.2% in 2020. Thereafter, the annual proportion increased and remained between 38.2% and 44.4% during 2021–2024. Overall, *Salmonella* spp. non-compliance remained frequent during the later years of surveillance.

### 3.4. Seasonal Patterns of Microbiological Non-Compliance

**Figure 4:**
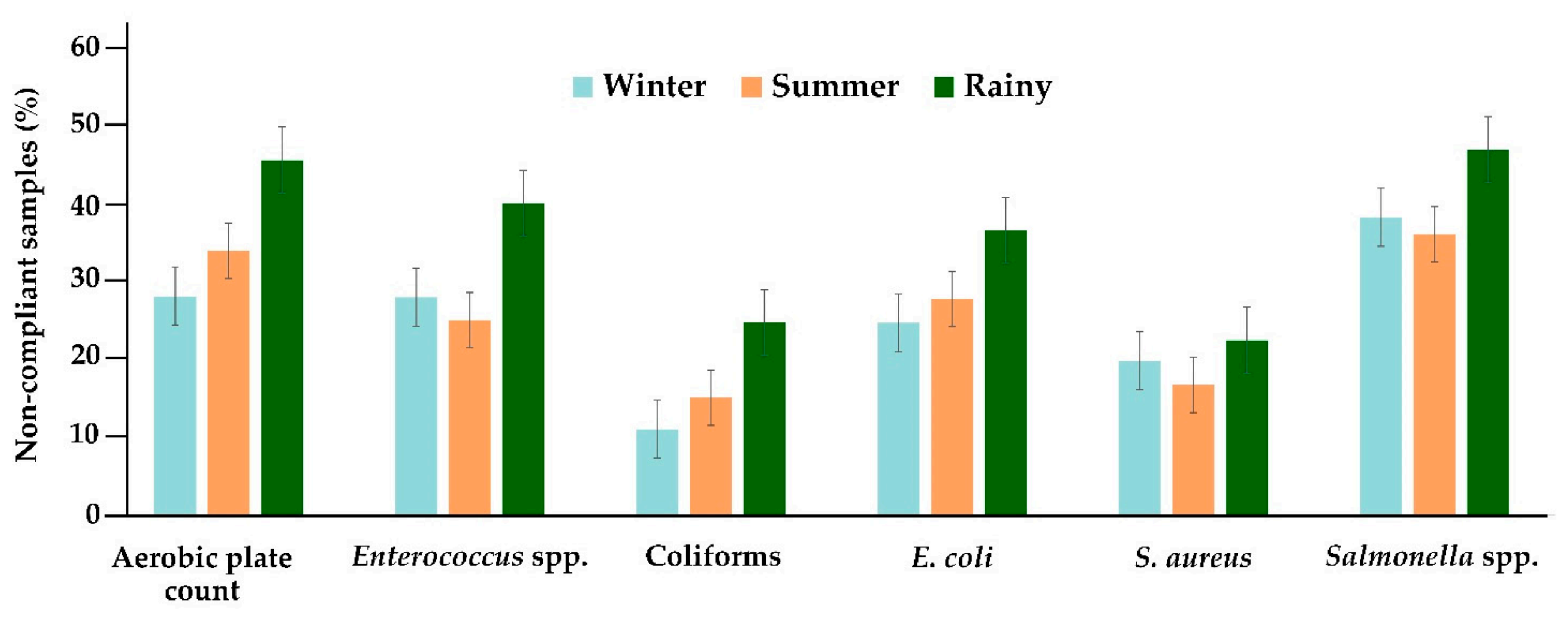
Proportions of non-compliant pork samples by season and microbiological endpoint in slaughterhouses in Lower Northern Thailand, 2015–2024. Bars represent the percentage of non-compliant samples among all samples tested within winter (n = 944), summer (n = 970), and the rainy season (n = 1189). Error bars represent 95% confidence intervals.

Seasonal variation in the proportions of non-compliant pork samples was observed across microbiological endpoints (Figure 4). In crude seasonal summaries, the rainy season had the highest non-compliance proportion for APC, coliforms, *E. coli*, *Enterococcus* spp., *S. aureus*, and *Salmonella* spp. For APC, coliforms, and *E. coli*, non-compliance increased from winter to summer and was highest during the rainy season. In contrast, the proportion of *Enterococcus* spp.-non-compliant samples was slightly lower in summer than in winter and was highest during the rainy season. Seasonal differences in *S. aureus* non-compliance were comparatively small, although the highest proportion was also observed during the rainy season. For *Salmonella* spp., non-compliance was lower in summer than in winter and highest during the rainy season.

Outcome-specific multivariable mixed-effects logistic regression models were adjusted for calendar year, slaughterhouse production scale, and province, with slaughterhouse ID included as a random intercept. Compared with winter samples, summer samples had lower odds of *Enterococcus* spp. non-compliance (AOR = 0.64, 95% CI: 0.48–0.86; *p* = 0.003). No statistically significant associations were observed between summer and non-compliance for APC, coliforms, *E. coli*, *S. aureus*, or *Salmonella* spp. In contrast, rainy-season samples had higher odds of coliform non-compliance (AOR = 1.75, 95% CI: 1.23–2.50; *p* = 0.002) and *Salmonella* spp. non-compliance (AOR = 1.51, 95% CI: 1.20–1.91; *p* < 0.001) than winter samples. Associations between the rainy season and APC, *E. coli*, *Enterococcus* spp., and *S. aureus* non-compliance were not statistically significant (Table 3).

### 3.5. Production-Scale Patterns of Non-Compliance

**Figure 5:**
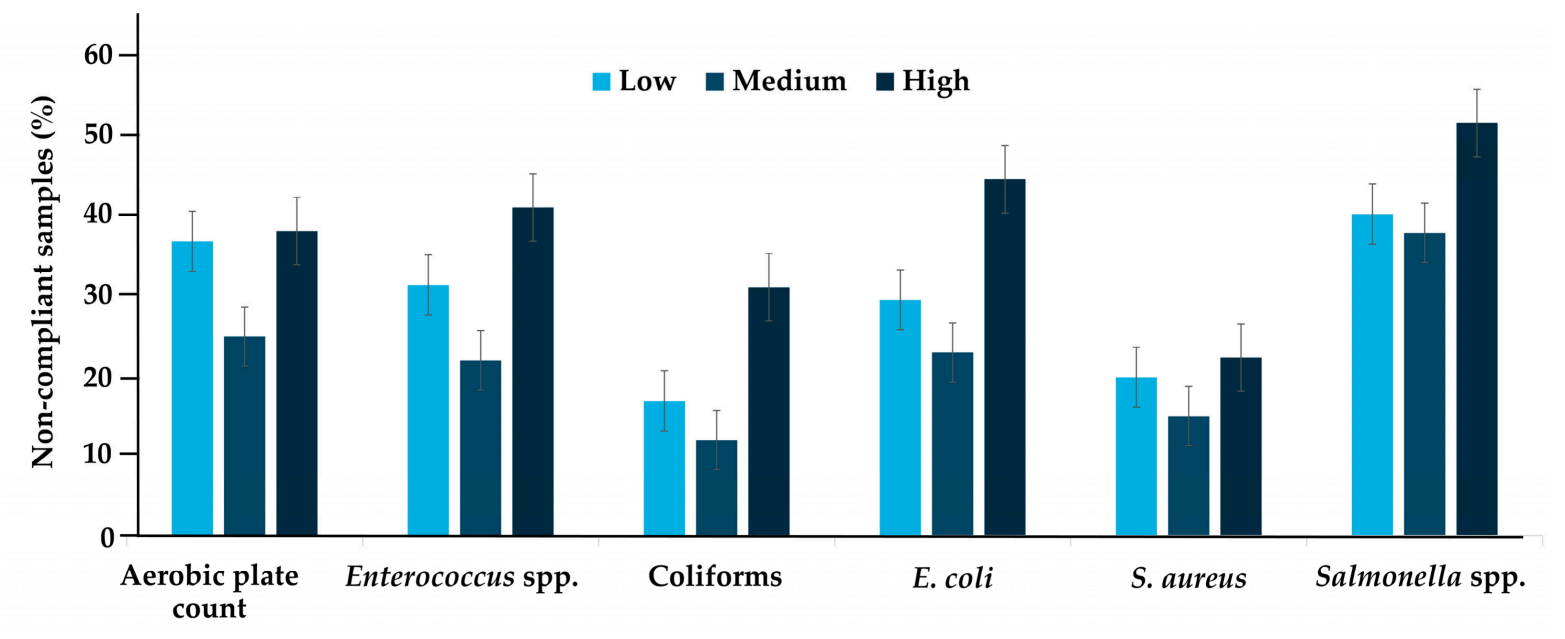
Proportions of non-compliant pork samples by slaughterhouse production scale and microbiological endpoint in Lower Northern Thailand, 2015–2024. Bars represent the percentage of non-compliant samples in low-, medium-, and high-scale slaughterhouses for each microbiological endpoint. Error bars represent 95% confidence intervals.

Descriptive differences in microbiological non-compliance proportions were observed across slaughterhouse production scales (Figure 5). High-scale slaughterhouses had the highest observed proportions of non-compliant samples for APC, coliforms, *E. coli*, *Enterococcus* spp., *S. aureus*, and *Salmonella* spp. In contrast, medium-scale slaughterhouses had the lowest observed proportions across all endpoints, whereas low-scale slaughterhouses generally had intermediate proportions. The corresponding distributions of slaughterhouses, pork samples, and non-compliant samples by production scale are presented in Supplementary Table S2.

Outcome-specific multivariable mixed-effects logistic regression models were adjusted for calendar year, season, and province, with slaughterhouse ID included as a random intercept. Using low-scale slaughterhouses as the reference category, high-scale slaughterhouses had higher odds of coliform non-compliance (AOR = 2.61, 95% CI: 1.21–5.64; *p* = 0.015), *E. coli* non-compliance (AOR = 2.39, 95% CI: 1.22–4.69; *p* = 0.011), and *Salmonella* spp. non-compliance (AOR = 2.02, 95% CI: 1.17–3.48; *p* = 0.012) (Table 4). No statistically significant adjusted associations were observed between high-scale slaughterhouses and APC, *Enterococcus* spp., or *S. aureus* non-compliance. Medium-scale slaughterhouses were not significantly associated with non-compliance for any microbiological endpoint, although a lower odds estimate was observed for *S. aureus* non-compliance (AOR = 0.41, 95% CI: 0.16–1.06; *p* = 0.066). Because the distribution of slaughterhouses across production-scale categories was highly unbalanced, production-scale estimates were interpreted cautiously.

A sensitivity analysis comparing high-scale with non-high-scale slaughterhouses showed consistent findings. High-scale slaughterhouses remained associated with higher odds of coliform, *E. coli*, and *Salmonella* spp. non-compliance, whereas APC, *Enterococcus* spp., and *S. aureus* were not significantly associated with high-scale status (Supplementary Table S4).

All outcome-specific mixed-effects logistic regression models converged without optimizer warnings, and no singular fits were detected. Slaughterhouse-level random-intercept variances ranged from 0.294 to 0.736, corresponding to ICC values of 0.082 to 0.183 on the logistic latent scale. Model diagnostics are presented in Supplementary Table S5.

## 4. Discussion

This 10-year surveillance study showed substantial variation in microbiological non-compliance among pork samples collected from slaughterhouses in Lower Northern Thailand. Although aerobic plate count (APC) had the highest median quantitative bacterial counts, *Salmonella* spp. was the most frequent non-compliance endpoint, with 40.90% of samples classified as non-compliant (Table 2). APC values extended to approximately 7.5 log_10_ CFU/g, indicating markedly elevated microbial loads in a subset of samples relative to the DLD criterion of 5.70 log_10_ CFU/g. These elevated values may reflect deficiencies in hygiene or handling at one or more stages of slaughter and processing, although the surveillance data did not permit identification of the specific contamination source. Taken together, the high frequency of *Salmonella* spp. non-compliance and the occurrence of markedly elevated APC values indicate that microbiological control remained an important concern within the surveillance system. These findings should be interpreted in relation to the different purposes of the microbiological endpoints. APC reflects the general microbial condition of a sample and includes a broad range of aerobic flora, including organisms that are not necessarily pathogenic, whereas coliforms, *Escherichia coli*, and *Enterococcus* spp. provide complementary information on fecal contamination, sanitation, and process hygiene [46,47]. In contrast, detection of *Salmonella* spp. indicates the presence of a recognized foodborne hazard, although the surveillance method used in this study did not characterize serovar, virulence determinants, or antimicrobial resistance profiles [48,49]. The present findings therefore describe *Salmonella* non-compliance at the genus level and should not be interpreted as evidence of equivalent virulence or antimicrobial-resistance potential among detected strains. Accordingly, compliance with hygiene-indicator criteria cannot be assumed to indicate the absence of *Salmonella* spp. The broad distributions and high outlying values observed for the quantitative endpoints (Figure 2) indicate considerable sample-level heterogeneity; however, the surveillance records did not identify the point of contamination or the slaughter and processing conditions associated with individual results. The findings therefore support the continued inclusion of both hygiene indicators and direct pathogen testing in routine pork surveillance. A nationwide Thai study applying the same DLD microbiological criteria also reported *Salmonella* spp. as the most frequent individual non-compliance endpoint, although direct numerical comparisons are limited by differences in geographic coverage, participating facilities, and sampling composition [21].

Crude non-compliance proportions also differed substantially among provinces (Table 2). Nakhon Sawan had the highest proportions of non-compliance for coliforms, *E. coli*, *Enterococcus* spp., and *Salmonella* spp., whereas Sukhothai had the lowest proportions for APC, coliforms, and *Enterococcus* spp. Lower non-compliance for hygiene-associated endpoints did not, however, consistently correspond to lower *Salmonella* spp. non-compliance. Geographic variation in pork contamination has also been reported in Thailand. A nationwide survey conducted in 2019 found that the northern region had the highest *Salmonella* spp. non-compliance among the six regions evaluated [21], while earlier work reported variation in *Salmonella* contamination among individual pig slaughterhouses in Chiang Mai and Lamphun [50]. The provincial patterns observed here should not be interpreted as causal effects of province. Participating slaughterhouses and sample numbers varied by year and province, and the dataset did not include information on pig origin, animal movement, transport conditions, lairage, water quality, slaughter practices, sanitation procedures, or inspection intensity. In addition, a supply-chain network analysis conducted in Nakhon Sawan did not evaluate *Salmonella* contamination and therefore cannot explain the higher non-compliance observed in the present study [51]. Nevertheless, consistently elevated non-compliance across fecal indicators and *Salmonella* spp. may be useful for identifying provinces or facilities requiring more detailed follow-up. Such investigations should focus on recurrent facility-level results and collect process-specific information on pig sourcing, lairage, water management, carcass handling, equipment sanitation, and opportunities for cross-contamination [48,49].

Annual non-compliance varied by microbiological endpoint and did not follow a common linear trend over the 10-year period (Figure 3). Non-compliance for APC, coliforms, *E. coli*, *Enterococcus* spp., and *S. aureus* was generally higher during 2015–2018 than in later years, although peak years differed among endpoints. APC non-compliance peaked in 2016, coliform, *E. coli*, and *Enterococcus* spp. non-compliance peaked in 2017, and *S. aureus* non-compliance was highest in 2015. Several hygiene-associated endpoints declined markedly between 2018 and 2019 and reached their lowest observed annual levels during 2021–2022. These changes occurred during the implementation periods of the DLD Strategy 2018–2022, the National Monitoring Program for Slaughterhouse Inspection and Certification, Thailand’s National Strategic Plan on Antimicrobial Resistance, and intensified animal-health and biosecurity activities associated with African swine fever preparedness [21,37,52,53]. The lowest observed levels for several endpoints also coincided with the COVID-19 pandemic and the period surrounding the first confirmed occurrence of African swine fever in Thailand [54,55,56]. These temporal overlaps provide institutional and epidemiological context, but they do not demonstrate that the observed changes were caused by any specific policy, disease event, or control program. Facility-level implementation, inspection frequency, production volume, slaughter practices, and changes in the population of participating slaughterhouses were not available for analysis.

Interpretation of the annual patterns should also account for variation in surveillance coverage. The number and identity of participating slaughterhouses differed across years according to annual surveillance plans; therefore, changes in annual non-compliance may partly reflect changes in the composition of sampled facilities rather than changes within the same slaughterhouses over time. According to information provided by the responsible authority, the microbiological criteria and analytical procedures remained unchanged during 2015–2024, except for the transition in *Salmonella* testing from ISO 6579:2002 to ISO 6579-1:2017. Nevertheless, detailed information on the exact timing and laboratory-level implementation of this methodological transition was unavailable. Annual differences should therefore be interpreted as surveillance patterns rather than direct estimates of changes in the underlying slaughterhouse population.

Seasonal associations differed across microbiological endpoints. Although the rainy season showed the highest unadjusted proportion of non-compliance for all endpoints (Figure 4), statistically significant adjusted associations were observed only for coliforms and *Salmonella* spp., which had higher odds during the rainy season than winter. Samples collected during summer had lower adjusted odds of *Enterococcus* spp. non-compliance than those collected during winter; however, this association should not be interpreted as evidence that summer conditions directly reduce contamination. Season represents a combination of environmental and operational conditions that were not measured individually in this surveillance dataset. In particular, meteorological conditions, water quality, drainage performance, cleaning practices, and the microbiological status of incoming pigs were unavailable, and the mechanisms underlying the observed associations could not be determined. A study conducted in Cambodian markets similarly reported a higher wet-season prevalence of *Salmonella* spp. in chicken, pork, and cutting-board samples [57], although those findings were generated at the retail stage and cannot be directly extrapolated to slaughterhouse surveillance. The present results therefore support interpreting season as a marker of broader environmental and operational variation rather than as an independent causal determinant. During the rainy season, closer review of drainage, water management, cleaning effectiveness, and evisceration hygiene may complement microbiological monitoring, particularly where coliform or *Salmonella* spp. non-compliance recurs.

The unadjusted proportion of microbiological non-compliance varied across low-, medium-, and high-scale slaughterhouses (Figure 5). Medium-scale slaughterhouses had the lowest crude proportion across endpoints, whereas high-scale facilities generally had the highest. After adjustment for calendar year, season, province, and slaughterhouse-level clustering, high-scale slaughterhouses had higher odds of coliform, *E. coli*, and *Salmonella* spp. non-compliance than low-scale facilities (Table 4). In contrast, no statistically significant adjusted associations were observed for APC, *Enterococcus* spp., or *S. aureus*, and medium-scale slaughterhouses were not significantly associated with non-compliance for any endpoint. These findings should therefore be interpreted as endpoint-specific associations and not as evidence that one production-scale category is uniformly safer or more hazardous than another. Interpretation of the production-scale findings also requires consideration of the markedly unbalanced distribution of facilities, as low-scale slaughterhouses accounted for the great majority of establishments represented in the study (Supplementary Table S2). Because relatively few medium- and high-scale slaughterhouses contributed data, estimates for these categories may be influenced by the characteristics of a small number of facilities, including sourcing patterns, throughput, sanitation practices, and other unmeasured operating conditions. The lack of statistically significant associations for medium-scale facilities should therefore not be interpreted as evidence of lower risk, particularly given the limited number of facilities in this category. Previous studies have likewise reported inconsistent relationships between slaughterhouse capacity and microbiological contamination, with some identifying higher *Salmonella* occurrence in medium-capacity facilities and others finding no clear difference by abattoir size [21,27,58].

The higher adjusted odds of coliform, *E. coli*, and *Salmonella* spp. non-compliance in high-scale slaughterhouses may reflect characteristics associated with greater throughput and operational complexity, such as line speed, carcass handling practices, equipment use, and opportunities for cross-contamination [42,43,59]. However, none of these process-level factors was measured directly, and production scale should therefore be regarded as a proxy for a broader set of facility characteristics rather than as a causal determinant of non-compliance. The sensitivity analysis compared high-scale slaughterhouses with all other facilities by combining low- and medium-scale slaughterhouses into a non-high-scale reference group. The resulting estimates were similar to those from the primary analysis (Supplementary Table S4). Nevertheless, the limited number of high-scale slaughterhouses and the absence of process-level data preclude identification of the specific conditions responsible for the observed associations. From a surveillance perspective, the findings support prioritizing follow-up assessments in facilities with recurrent coliform, *E. coli*, or *Salmonella* spp. non-compliance rather than applying assumptions based on production scale alone. Linking microbiological results with standardized information on throughput, line speed, water use, sanitation procedures, evisceration practices, and corrective actions would allow future analyses to assess whether the observed production-scale associations are explained by specific operational conditions.

Several limitations should be considered. First, the routine surveillance design did not permit identification of specific contamination sources, and the lack of farm-, animal-, transport-, and process-level information meant that the mechanisms proposed to explain the observed associations could not be evaluated directly. Second, medium- and high-scale slaughterhouses were represented by relatively few facilities, which limited the precision and generalizability of estimates for these categories. Although the sensitivity analysis produced estimates similar to those of the primary analysis when high-scale slaughterhouses were compared with a combined non-high-scale group (Supplementary Table S4), it does not exclude the influence of facility-specific characteristics or other unmeasured factors. Third, surveillance sampling was not probability-based, and the slaughterhouses contributing samples varied across years and provinces. Consequently, observed differences in sample-level non-compliance may partly reflect changes in surveillance coverage and facility composition rather than microbiological conditions alone. Residual confounding is therefore possible, and the findings should be interpreted as associations observed within the surveillance data rather than causal effects or definitive measures of slaughterhouse performance.

Despite these limitations, the long surveillance period, large sample size, multi-province coverage, and use of outcome-specific mixed-effects models provided a useful basis for identifying recurring patterns of microbiological non-compliance under routine surveillance conditions. The results support considering season and production scale as components of risk-based surveillance, but not as stand-alone indicators of contamination risk. During the rainy season, surveillance findings for coliforms and *Salmonella* spp. could be used to guide closer review of drainage, water management, cleaning effectiveness, evisceration hygiene, and separation of clean and dirty areas. Similarly, high-scale slaughterhouses with recurrent coliform, *E. coli*, or *Salmonella* spp. non-compliance may warrant facility-level follow-up, while avoiding assumptions that all high-scale facilities are at greater risk. Facilities or provinces with repeated *Salmonella* spp. non-compliance should be considered for further investigation of pig sourcing, transport and lairage conditions, carcass handling, sanitation practices, and possible environmental reservoirs. Linking future microbiological surveillance with standardized process, inspection, and corrective-action records would improve the ability to distinguish recurrent facility-level problems from variation caused by sampling coverage or unmeasured operating conditions. This approach may also be relevant to other tropical settings with heterogeneous slaughterhouse systems and seasonally variable operating conditions.

## 5. Conclusions

This 10-year surveillance study examined 3103 pork samples collected from slaughterhouses in Lower Northern Thailand during 2015–2024. *Salmonella* spp. was the most frequent non-compliance endpoint (40.90%) and remained common throughout the surveillance period, including during years when several hygiene-associated endpoints had lower non-compliance. The observed associations differed across microbiological endpoints. Rainy-season samples had higher odds of coliform and *Salmonella* spp. non-compliance than winter samples, whereas high-scale slaughterhouses had higher odds of coliform, *E. coli*, and *Salmonella* spp. non-compliance than low-scale facilities. These findings should be interpreted as associations within routine surveillance data rather than causal effects, because contamination sources, processing conditions, and facility-level mechanisms were not directly measured. Overall, the results indicate that hygiene indicators should not be used as a substitute for direct pathogen testing and support risk-based surveillance that combines microbiological results with standardized information on animal sourcing, slaughter processes, sanitation, environmental conditions, and corrective actions. Such integration could improve the identification of recurrent non-compliance and guide targeted follow-up across the farm-to-slaughterhouse continuum.

## Figures and Tables

**Table 1:** Microbiological criteria and reference methods used for classification of pork samples as compliant or non-compliant.

Microbiological Endpoint	Criteria *	Reference
Aerobic plate count	≤5.70 log_10_ CFU/g	FDA BAM Online, 2001 (Chapter 3) [39]
Coliforms	≤3.70 log_10_ CFU/g	FDA BAM Online, 2013 (Chapter 4) [40]
*Escherichia coli*	≤2.00 log_10_ CFU/g	FDA BAM Online, 2013 (Chapter 4) [40]
*Enterococcus* spp.	≤3.00 log_10_ CFU/g	Nordic Committee on Food Analysis, No. 68 5th Edition (2011) [41]
*Staphylococcus aureus*	≤2.00 log_10_ CFU/g	ISO 6888-1:1999 [42]
*Salmonella* spp.	Not detected in 25 g of sample	ISO 6579:2002 [44]; ISO 6579-1:2017 [43]

* Microbiological Criteria of the Department of Livestock Development, Thailand.

**Table 2:** Proportions of microbiological non-compliance in pork samples by province in Lower Northern Thailand, 2015–2024.

Provinces	Non-Compliant Pork Samples, % (n)					
	Aerobic Plate Count	Coliforms	*Escherichia coli*	*Enterococcus* spp.	*Staphylococcus aureus*	*Salmonella* spp.
Phichit (N = 574)	43.03 (247)	18.12 (104)	29.27 (168)	33.97 (195)	15.33 (88)	42.16 (242)
Phitsanulok (N = 672)	37.50 (252)	20.09 (135)	28.57 (192)	29.61 (199)	19.94 (134)	37.2 (250)
Uttaradit (N = 250)	23.20 (58)	5.20 (13)	12.40 (31)	15.20 (38)	14.40 (36)	35.20 (88)
Sukhothai (N = 168)	18.45 (31)	4.76 (8)	19.05 (32)	14.88 (25)	19.64 (33)	39.29 (66)
Tak (N = 99)	45.45 (45)	19.19 (19)	36.36 (36)	32.32 (32)	16.16 (16)	43.43 (43)
Kamphaeng Phet (N = 229)	21.83 (50)	19.65 (45)	24.02 (55)	30.13 (69)	22.27 (51)	34.93 (80)
Phetchabun (N = 329)	36.47 (120)	15.81 (52)	35.26 (116)	32.83 (108)	23.71 (78)	40.43 (133)
Nakhon Sawan (N = 365)	44.38 (162)	26.58 (97)	46.58 (170)	42.47 (155)	21.92 (80)	53.42 (195)
Uthai Thani (N = 417)	41.01 (171)	17.03 (71)	32.85 (137)	38.85 (162)	23.98 (100)	41.25 (172)
Total (N = 3103)	36.61 (1136)	17.53 (544)	30.20 (937)	31.68 (983)	19.85 (616)	40.90 (1269)

*Note.* Data are presented as % (n), where n is the number of non-compliant pork samples. N indicates the total number of pork samples examined within each province.

**Table 3:** Adjusted associations between season and microbiological non-compliance in pork samples: outcome-specific multivariable mixed-effects logistic regression models.

Microbiological Endpoint	Summer Season vs. Winter AOR (95% CI)	*p*-Value	Rainy Season vs. Winter AOR (95% CI)	*p*-Value
Aerobic plate count	0.94 (0.70–1.25)	0.649	1.26 (0.97–1.65)	0.084
Coliforms	1.08 (0.73–1.59)	0.706	1.75 (1.23–2.50)	0.002
*Escherichia coli*	0.99 (0.74–1.33)	0.931	1.17 (0.90–1.53)	0.244
*Enterococcus* spp.	0.64 (0.48–0.86)	0.003	1.11 (0.85–1.44)	0.458
*Staphylococcus aureus*	0.93 (0.65–1.34)	0.708	1.01 (0.73–1.38)	0.964
*Salmonella* spp.	1.16 (0.91–1.49)	0.238	1.51 (1.20–1.91)	<0.001

*Note.* AOR, adjusted odds ratio; CI, confidence interval. Separate outcome-specific multivariable mixed-effects logistic regression models were fitted for each microbiological endpoint. Models included calendar year, season, slaughterhouse production scale, and province as fixed effects, with slaughterhouse ID included as a random intercept. Reference categories were 2015 for calendar year, winter for season, low-scale slaughterhouses for production scale, and Phichit for province.

**Table 4:** Adjusted associations of slaughterhouse production scale with microbiological non-compliance in pork samples: outcome-specific multivariable mixed-effects logistic regression models.

Microbiological Endpoint	Medium-Scale vs. Low-Scale AOR (95% CI)	*p*-Value	High-Scale vs. Low-Scale AOR (95% CI)	*p*-Value
Aerobic plate count	0.90 (0.34–2.42)	0.835	1.23 (0.59–2.58)	0.580
Coliforms	1.39 (0.42–4.65)	0.591	2.61 (1.21–5.64)	0.015
*Escherichia coli*	1.06 (0.42–2.67)	0.899	2.39 (1.22–4.69)	0.011
*Enterococcus* spp.	1.16 (0.44–3.06)	0.763	1.61 (0.79–3.25)	0.187
*Staphylococcus aureus*	0.41 (0.16–1.06)	0.066	0.77 (0.37–1.61)	0.488
*Salmonella* spp.	0.77 (0.38–1.54)	0.458	2.02 (1.17–3.48)	0.012

*Note.* AOR, adjusted odds ratio; CI, confidence interval. Separate outcome-specific multivariable mixed-effects logistic regression models were fitted for each microbiological endpoint. Models included calendar year, season, slaughterhouse production scale, and province as fixed effects, with slaughterhouse ID included as a random intercept. Reference categories were 2015 for calendar year, winter for season, low-scale slaughterhouses for production scale, and Phichit for province.

## Data Availability

The data presented in this study are not publicly available because they were derived from routine surveillance activities of the Department of Livestock Development, Thailand, and contain facility-level information. Access to the data may be considered by the Department of Livestock Development, Thailand, subject to applicable data-sharing requirements.

## Supplementary Materials

The following supporting information can be downloaded at https://www.mdpi.com/article/10.3390/foods15172980/s1, Table S1: Annual distribution of surveillance visits, participating slaughterhouses, and pork samples by slaughterhouse production scale in Lower Northern Thailand, 2015–2024; Table S2: Distribution of slaughterhouses, pork samples, and microbiological non-compliance by slaughterhouse production scale in Lower Northern Thailand, 2015–2024; Table S3: Distribution of slaughterhouses and pork samples by production scale and province in Lower Northern Thailand, 2015–2024; Table S4: Sensitivity analysis of the association between slaughterhouse production scale and microbiological non-compliance; Table S5: Model diagnostics for outcome-specific multivariable mixed-effects logistic regression models.
