# Supplementary material for: Ten-Year Surveillance of Microbiological Non-Compliance in Pork: Associations with Season and Slaughterhouse Scale in Lower Northern Thailand
Source: Foods. 2026 Aug 25;15(17):2980. doi: 10.3390/foods15172980 (PMC13564512; doi:10.3390/foods15172980)
Supplement: Supplementary file 1 [file foods-15-02980-s001.zip › foods-4493828-supplementary.pdf]

Supplementary Materials

Table S1. Annual distribution of surveillance visits, participating slaughterhouses, and pork samples by slaughterhouse production scale in Lower Northern Thailand, 2015–2024.

Year	Pork samples, n	Surveillance visits, n	Participating slaughterhouses, n	Low-scale samples, n (%)	Medium-scale samples, n (%)	High-scale samples, n (%)
**2015**	286	155	106	232 (81.12%)	29 (10.14%)	25 (8.74%)
**2016**	212	120	95	194 (91.51%)	8 (3.77%)	10 (4.72%)
**2017**	288	199	164	256 (88.89%)	9 (3.12%)	23 (7.99%)
**2018**	620	591	322	588 (94.84%)	9 (1.45%)	23 (3.71%)
**2019**	509	504	278	481 (94.50%)	10 (1.96%)	18 (3.54%)
**2020**	352	343	296	337 (95.74%)	5 (1.42%)	10 (2.84%)
**2021**	136	135	135	128 (94.12%)	6 (4.41%)	2 (1.47%)
**2022**	186	186	181	179 (96.24%)	0 (0.00%)	7 (3.76%)
**2023**	264	248	148	224 (84.85%)	12 (4.55%)	28 (10.61%)
**2024**	250	237	152	215 (86.00%)	12 (4.80%)	23 (9.20%)

Note. A surveillance visit was defined as the collection of one or more pork samples from a single slaughterhouse on a given reporting date. Participating slaughterhouses were defined as unique facilities contributing at least one pork sample during the corresponding calendar year. The number and composition of participating slaughterhouses varied across years because surveillance was implemented under annual provincial operational plans. Percentages for slaughterhouse production-scale categories were calculated using the total number of pork samples collected in each calendar year as the denominator.

Table S2. Distribution of slaughterhouses, pork samples, and microbiological non-compliance by slaughterhouse production scale in Lower Northern Thailand, 2015–2024.

Variable	Production scale		
	Low	Medium	High
No. of slaughterhouses	394	6	11
No. of pork samples	2,833	100	169
APC non-compliant, n (%)	1,046 (36.91)	25 (25.00)	65 (38.46)
Coliform non-compliant, n (%)	479 (16.90)	12 (12.00)	53 (31.36)
E. coli non-compliant, n (%)	838 (29.57)	23 (23.00)	76 (44.97)
Enterococcus spp. non-compliant, n (%)	891 (31.44)	22 (22.00)	70 (41.42)
S. aureus non-compliant, n (%)	563 (19.87)	15 (15.00)	38 (22.49)
Salmonella spp. non-compliant, n (%)	1,143 (40.33)	38 (38.00)	88 (52.07)

Note. APC, aerobic plate count. Data are presented as n (%). Percentages for microbiological non-compliance were calculated using the number of pork samples within each production-scale category as the denominator. The number of slaughterhouses represents unique facilities contributing at least one pork sample during the surveillance period.

Table S3. Distribution of slaughterhouses and pork samples by production scale and province in Lower Northern Thailand, 2015–2024.

Characteristic	Category	Low-scale, n (%)	Medium-scale, n (%)	High-scale, n (%)	Total, n (%)
**Unique slaughterhouses**	—	394 (95.86)	6 (1.46)	11 (2.68)	411 (100.00)
**Pork samples**	—	2,833 (91.30)	100 (3.22)	170 (5.45)	3,103 (100.00)
**Province**	Phichit	574 (100.00)	0 (0.00)	0 (0.00)	574 (18.50)
	Phitsanulok	661 (98.36)	0 (0.00)	11 (1.64)	672 (21.66)
	Uttaradit	233 (93.20)	17 (6.80)	0 (0.00)	250 (8.06)
	Sukhothai	101 (60.12)	67 (39.88)	0 (0.00)	168 (5.41)
	Tak	47 (47.47)	0 (0.00)	52 (52.53)	99 (3.19)
	Kamphaeng Phet	148 (64.63)	0 (0.00)	81 (35.37)	229 (7.38)
	Phetchabun	319 (96.96)	0 (0.00)	10 (3.04)	329 (10.60)
	Nakhon Sawan	334 (91.51)	16 (4.38)	15 (4.11)	365 (11.76)
	Uthai Thani	417 (100.00)	0 (0.00)	0 (0.00)	417 (13.44)

Note. Data are presented as n (%). For the rows "Unique slaughterhouses" and "Pork samples," percentages indicate the distribution across production-scale categories. For province rows, percentages in the low-, medium-, and high-scale columns indicate the proportion of pork samples from each province contributed by each production-scale category, whereas percentages in the total column were calculated using all 3,103 pork samples as the denominator. The number of slaughterhouses represents unique facilities contributing at least one sample during the surveillance period.

Table S4. Sensitivity analysis of the association between slaughterhouse production scale and microbiological non-compliance.

Microbiological endpoint	Primary analysis: high-scale vs low-scale, AOR (95% CI)	p-value	Sensitivity analysis: high-scale vs non-high-scale, AOR (95% CI)	p-value
**APC**	1.23 (0.59–2.58)	0.580	1.22 (0.58–2.55)	0.601
**Coliforms**	2.61 (1.21–5.64)	0.015	2.58 (1.19–5.59)	0.016
E. coli	2.39 (1.22–4.69)	0.011	2.39 (1.22–4.69)	0.011
***Enterococcus* spp.**	1.61 (0.79–3.25)	0.187	1.61 (0.79–3.25)	0.187
S. aureus	0.77 (0.37–1.61)	0.488	0.78 (0.37–1.64)	0.514
***Salmonella* spp.**	2.02 (1.17–3.48)	0.012	2.02 (1.17–3.49)	0.011

Note. APC, aerobic plate count; AOR, adjusted odds ratio; CI, confidence interval. The primary analysis used low-scale slaughterhouses as the reference category. The sensitivity analysis compared high-scale slaughterhouses with non-high-scale slaughterhouses, defined as low- and medium-scale facilities combined. Separate outcome-specific multivariable mixed-effects logistic regression models were fitted for each microbiological endpoint. Models were adjusted for calendar year, season, and province, with slaughterhouse ID included as a random intercept.

Table S5. Model diagnostics for outcome-specific multivariable mixed-effects logistic regression models.

Model diagnostic	Aerobic plate count	Coliforms	*Escherichia coli*	*Enterococcus* spp.	*Staphylococcus aureus*	*Salmonella* spp.
**Model observations, n**	3,103	3,103	3,103	3,103	3,103	3,103
**Non-compliant samples, n (%)**	1,136 (36.61)	544 (17.53)	937 (30.20)	983 (31.68)	616 (19.85)	1,269 (40.90)
**Convergence warning**	No	No	No	No	No	No
**Singular fit**	No	No	No	No	No	No
**Random-intercept variance**	0.736	0.664	0.561	0.648	0.491	0.294
**Random-intercept SD**	0.858	0.815	0.749	0.805	0.701	0.542
**ICC**	0.183	0.168	0.146	0.165	0.130	0.082
**Marginal R^2**	0.139	0.188	0.119	0.150	0.141	0.046
**Conditional R^2**	0.296	0.324	0.248	0.290	0.253	0.123

Note. Each outcome-specific model included 3,103 pork samples. Non-compliant samples indicate the number and percentage of samples classified as non-compliant for each microbiological endpoint. ICC, intraclass correlation coefficient; SD, standard deviation. All models converged successfully without optimizer warnings, and no singular fits were detected. ICC was calculated on the logistic latent scale. Random-intercept variance and SD refer to the slaughterhouse-level random intercept. Marginal R^2 represents the variance explained by fixed effects, whereas conditional R^2 represents the variance explained by both fixed effects and the slaughterhouse-level random intercept.
